# INDETERMINATE DOMAIN 9 negatively regulates rice crown root development

**DOI:** 10.3389/fpls.2026.1820594

**Published:** 2026-04-13

**Authors:** Yuanyuan Yang, Zihao Shi, Xuan Nie, Yuehang Meng, Zhixun Chu, Wenjing Zou, Qingmei Jian, Deyan Zhu, Ting Zhang

**Affiliations:** 1 College of Food and Biology, Jingchu University of Technology, Jingmen, Hubei, China; 2 Hubei Engineering Research Center for Specialty Flowers Biological Breeding, Jingchu University of Technology, Jingmen, Hubei, China

**Keywords:** crown root, OsCRL5, OsIDD9, OsWOX11, rice, uORF

## Abstract

**Introduction:**

Crown root is an important part of rice root system, and the development of crown root is regulated by a series of plant-specific transcription factors. As a significant plant-specific transcription factor family, the relationship between IDD and rice crown root development remains elusive.

**Methods:**

Here the function of *OsIDD9* in rice crown root development was identified by genetic, biochemical, and molecular evidence.

**Results:**

*OsIDD9* over-expression lines exhibited a defective crown root phenotype, while *OsIDD9* knockout lines displayed vigorous crown roots, indicating that *OsIDD9* is a negative regulator of crown root development. Further studies have shown that *OsIDD9* affects crown root development by regulating the expression of downstream key genes such as *OsWOX11, OsCRL5*. In addition, the expression of *OsIDD9* is regulated by the upstream open reading frame (uORF) at translation level.

**Discussion:**

This study reveals the role of the *OsIDD9*-mediated genetic pathway in the development of rice crown roots, and provides theoretical support for clarifying the interactions among different transcription factors in rice crown root development.

## Introduction

Rice (*Oryza sativa*) is an important monocot plant with a robust fibrous root system. Crown root (CR) is the main component of its root system to absorb water and nutrients, and respond to various stresses (Seo et al., 2020). Numerous studies have shown that the CR development is complex and regulated by various factors such as hormones, transcription factors (TFs), and environmental conditions, which usually constitute complex networks centered on TFs (Mao et al., 2020; Pan et al., 2020; Gonin et al., 2022; Liu et al., 2023; Singh et al., 2023; Zhong et al., 2025). At present, TFs that have been reported to affect CR development are OsCRL1 (CROWN ROOTLESS 1), OsCRL5, OsNAC2 (NAM, ATAF and CUC 2), OsDOF11 (DNA BINDING WITH ONE FINGER 11), OsWOX11 (WUSCHEL-RELATED HOMEOBOX 11), and so on. OsCRL1 is one of the plant-specific ASYMMETRIC LEAVES2-LIKE/LATERAL ORGAN BOUNDARIES DOMAIN (ASL/LBD) family and identified as the first positive factor to regulate rice CR formation (Inukai et al., 2005). OsCRL5 belongs to AP2 (APETALA2)/ERF(EHYLENE RESPONSIVE FACTOR) family and is essential for CR development by upregulating *OsRR1* (*A-type response regulator 1*) expression to promote CR initiation (Kitomi et al., 2011). OsNAC2, a plant-specific TF, negatively regulates CR number by integrating auxin and cytokinin pathways (Mao et al., 2020). OsDOF11, which belongs to a plant-specific DOF (DNA binding with One Finger) family, promotes rice CR formation via cytokinin (Dong et al., 2022). OsWOX11 is a member of the plant-specific WOX (WUSCHEL-related homeobox) family. Functional analysis revealed that *OsWOX11* over-expression lines led to ectopic CR formations, while loss-of-function of *OsWOX11* exhibited fewer CR (Zhao et al., 2009). All of these demonstrate the importance of plant-specific TFs in rice CR development.

The *INDETERMINATE DOMAIN* (*IDD*) gene also encodes a plant-specific TF and has been reported to be widely involved in plant growth and development and stress tolerance (Coelho et al., 2018; Kumar et al., 2019; Kozaki, 2024). Although the functional information of *OsIDD*s in rice has limited than that in *Arabidopsis*, recent studies have revealed several essential functions of *OsIDD*s. OsID1/Rice Indeterminate 1 (RID1), an ortholog of maize INDETERMINATE 1 (ZmID1), has been identified as the master regulator for the promotion of flowering. Functional analysis demonstrated that *OsID1* mutant (*rid1*) exhibited a never-flowering phenotype (Wu et al., 2008). Subsequent research suggested that over-expression of *OsID4* (*Suppressor of rid1*, *SID1*), *OsIDD1*, or *OsIDD6* in *rid1* can restore flowering of *rid1* (Deng et al., 2017). *OsIDD2* over-expressing plants showing severe dwarfism, fragile leaves, and decreased lignin content suggested OsIDD2 was a negative factor in secondary cell wall formation and other biological events (Huang et al., 2018). OsIDD3 can function as a positive regulator for chilling tolerance by upregulating the expression of *OsCBF1* (*C-repeat binding factor*) and *OsCBF3* (Dou et al., 2016). OsIDD10 play a vital role in nitrogen regulatory circuits that influence rice root growth (Xuan et al., 2013). OsIDD12 and OsIDD13 suppress *OsPIN5c* (*PIN FORMED5c*) expression by interacting with OsSHRs (SHORT ROOT) to regulate rice vein patterning (Sun et al., 2020). OsIDD14/Loose Plant Architecture1 (LPA1) was reported as a transcription repressor and involved in rice plant architecture formation. On the other hand, OsIDD3, OsIDD13, OsIDD14 physically interacted with and formed a transcription complex to modulate the resistance of rice to Sheath blight disease (Sun et al., 2020). Overall, these reported *OsIDD* genes mainly function in rice nutrient uptake and above-ground morphogenesis, while the role of *OsIDD* genes in rice root development remains unclear. In our previous analysis of *OsIDD* gene family, *OsIDD9* was highly expressed in roots (Zhang et al., 2020), and it was speculated to be related with rice root development. Therefore, it is necessary to clarify whether *OsIDD9* regulates rice root development and its relationship with other TFs regulating rice root development.

Here, the function of *OsIDD9* in rice CR development was explored. We observed that the CR number increased in the *OsIDD9* mutant (*osidd9*), while *OsIDD9* over-expression (*OE*) plants had fewer CRs. Moreover, the expression level of genes related to CR development such as *OsWOX11*, *OsCRL5* increased in *osidd9*. More interestingly, an active uORF, one kind of translation regulatory elements (Kurihara, 2020; Um et al., 2021; Wang et al., 2024), was found within the 5’UTR of *OsIDD9*. Taken together, these results reveal that *OsIDD9* is involved in rice CR development by negatively regulating the expression level of *OsWOX11* and *OsCRL5*, and its own expression may be regulated at the translation level. These will help to further improve the regulatory network of rice CR development centered on TFs.

## Materials and methods

### Bioinformatics analysis

Physicochemical properties (PIs, MWs) of OsIDD9 protein were predicted through the ExPASy website (https://www.expasy.org/) (Larkin et al., 2007). The amino acid sequences of OsIDD and AtIDD proteins were harvested from the Rice Genome Annotation Project Database (RGAP, https://rice.uga.edu/) and the TAIR10 database (https://www.arabidopsis.org/), respectively. After multiple sequence alignment using Clustal X (Wilkins et al., 1999), a neighbor-joining (NJ) tree was constructed with the Poisson model and 1, 000 replicates as the construction parameters using MEGA 7.0 software (Kumar et al., 2016).

### Plant materials and growth conditions

The rice materials used in this experiment were all ZH11 (Zhonghua 11, *Oryza sativa* ssp *Japonica*/*geng*), and the materials were cultured in the greenhouse (28 °C, 16 h) of the College of Food and Biology in Jingchu University of Technology. Seeds were harvested, dried and kept in a low humidity seed preservation cabinet (BIOFUTURE, HZDs-400). Tobacco materials used in this experiment were *Nicotiana benthamiana*, which was grown at 28 °C (in the light) and 24 °C (in the dark) in a light culture room.

### Vector construction

For *osidd9* knockout lines, two target sites of *OsIDD9* gene (GTATTACAGCAAGCTGCTCCTGG and TTCTTCTTCTTCACGGCCGTCGG) were selected and designed as sgRNA (single-guide RNA) using the CRISPR-P program (http://crispr.hzau.edu.cn/CRISPR2/). The CRISPR-cas9-mediated gene editing constructs were constructed according to He et al (He et al., 2018). For *OsIDD9* over-expression, the full-length cDNA of *OsIDD9* was amplified with the OsIDD9-FLAG-F and OsIDD9-FLAG-R primer set and inserted into the pU2301-3×FLAG vector (Sun and Zhou, 2008). To produce the OsIDD9 protein, the full-length cDNA amplified with primers IDD9-32a-F/R was inserted into the pET-32a vector, expressed in E.coli BL21, and purified using His-beads (QIAGEN, 30230). The 35S:uORF_IDD9_-LUC and 35S:uorf_IDD9_-LUC vectors were constructed in the vector pGX-5Dual provided by Prof. Dr. Guoyong Xu (Wuhan University, Wuhan, China). The 5’UTR containing uORF_IDD9_ was harvested by amplifying ZH11 cDNA with the uORF_IDD9_-LUC-F and uORF_IDD9_-LUC-R primer set and then cloned into pGX-5Dual. The 5’UTR containing uorf_IDD9_ was synthesized by Sangon Biotech. The primers were listed in **Supplementary Table 1**.

### Protein extraction and immunoblotting

Take about 100 mg rice root, make its surface dry, and grind it into powder, which was added to extraction buffer and mixed well. After that, these samples were centrifuged at 12, 000 rpm for 10 min at 4°C and the supernatant was retained. The proteins in the supernatant were separated by 10% SDS-PAGE gel and then transferred onto a PVDF membrane. Immunoblotting was performed with an anti-FLAG (1:1, 000 dilution).

### Quantitative RT-PCR

To investigated the expression level of CR development-related genes and transient expression of *LUC* in tobaccos, RNA of these materials were extracted according to the manufacturer’s protocol of TransZol (Transgen ET101-01) and the concentration was verified by (). After reverse transcription reaction, qRT-PCR analysis for *OsIDD9*, *OsWOX11*, *OsCRL1*, *OsRR2*, *OsCKX4*, *OsLBD16*, *OsCRL5*, and *LUC* expression was carried out on a QuantStudio 6 Flex real-time PCR instrument (Applied Biosystems) using ChamQ SYBR^®^ qPCR Master Mix (Vazyme Q311-02). For each sample, three replicate reactions were performed. With *OsActin* as an internal control, the relative expression level of each gene as analyzed using the 2 ^−ΔΔCt^ method (Livak & Schmittgen, 2001) and was normalized (WT set to 1.0). The primers were listed in **Supplementary Table 1**.

### Dual-luciferase transient expression assay

For tobacco leaf transient transformation, the concentration of transformed *EHA105* harboring the *35S:uORF_IDD9_-LUC* or *35S:uorf_IDD9_-LUC* constructs was adjusted to the same level (OD_600_ = 0.1). Then, two types of EHA105 suspensions were injected into two separate parts in the same young tobacco leaf using a micro-injector. After 2 days of culture in a light culture room, tobacco leaves were used to observe the activity of cytosol-synthesized firefly luciferase (LUC) (Xu et al., 2017). For the transient transformation of rice protoplasts, the reporter plasmid (3 μg), the effector plasmid (3 μg), and the internal control plasmid (0.5 μg) were mixed and introduced into suspension-cultured rice protoplasts. Transformed rice protoplasts were incubated for 12 h at 24 °C in the dark. The quantification of LUC and Renilla luciferase (REN) activities in transient transformation was performed by using the Dual-Luciferase^®^ Reporter Assay Kit (Promega, E1910).

### Electrophoretic mobility shift assay

Single-stranded complementary oligonucleotide fragments containing the IDD binding motifs were synthesized and labeled with biotin (Sangon Biotech). Biotin-labeled and unlabeled oligonucleotide pairs were annealed as double-stranded probes after denaturation. After the DNA binding reaction of the manufacturer’s instructions (the LightShift^®^ Chemiluminescent EMSA Kit 20148, Thermo Fisher), electrophoresis was performed. Then, the protein-probe mixture and free probe were transferred to a nylon membrane and detected using the Chemiluminescent Nucleic Acid Detection Module (Thermo Fisher, 89880).

### Statistical analysis

Three biological replicates were performed for each experiment. Data was shown as the means (± SD) of three independent experiments. Statistical significance was analyzed by Student’s t‐test and One-way ANOVA in Prism v9.5.1.733.

## Results

### *OsIDD9* gene characterization

**Figure 1:**
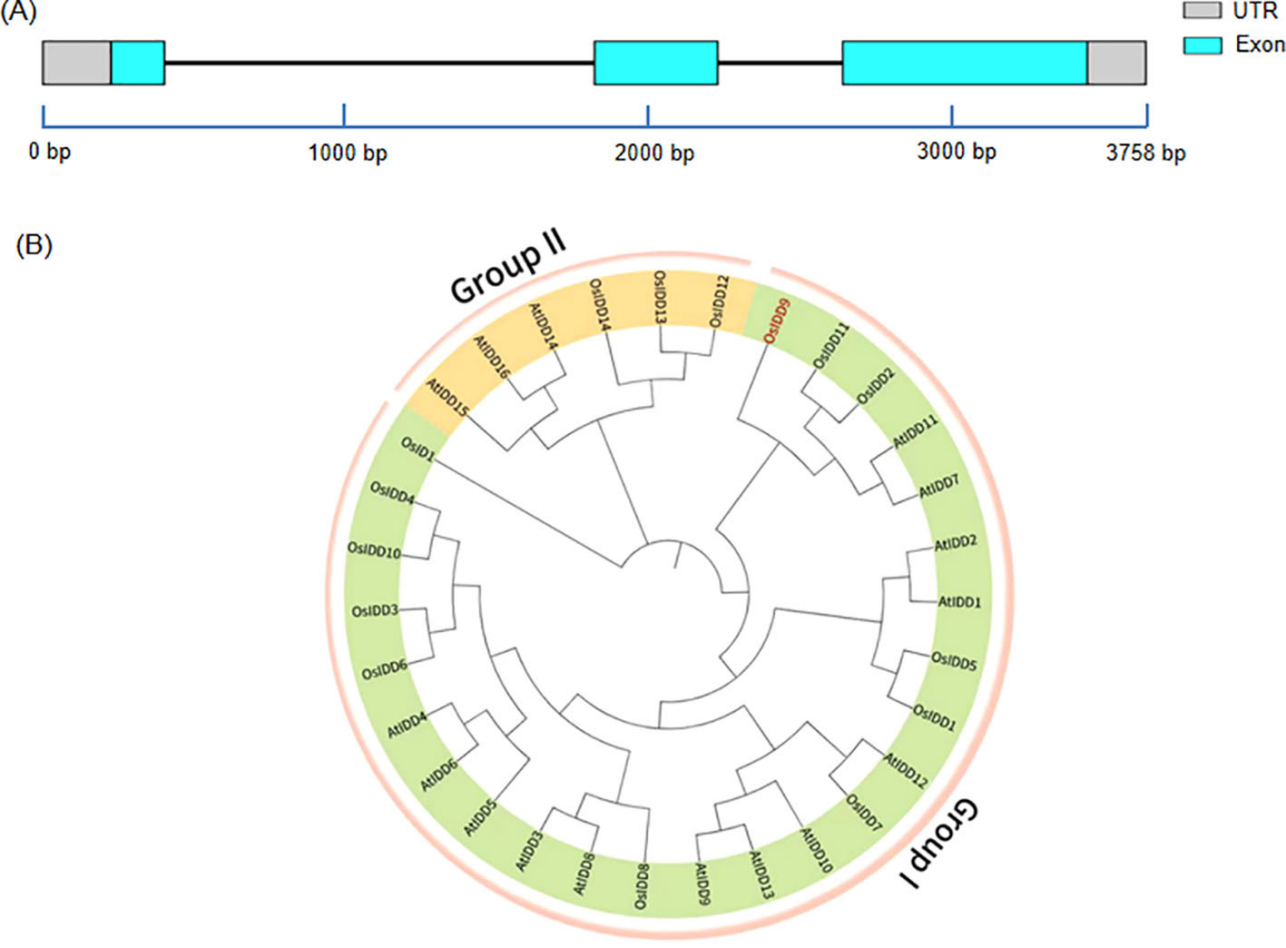
*OsIDD9* characterization and phylogenetic analysis. **(A)** Grey and blue blocks represented the untranslated regions (UTRs) and exons, respectively. Black lines indicated introns. **(B)** Phylogenetic analysis of OsIDD proteins in rice (*Oryza sativa*) and *Arabidopsis thaliana*. All protein sequences were obtained from Phytozome (https://phytozome.jgi.doe.gov/). Based on the sequence alignment result of IDDs by Clustal X, the neighbor-joining (NJ) tree was constructed using the MEGA 7.0 program with the default parameters (Poisson model, complete deletion, 1000 bootstrap replicates).

The *OsIDD9* sequence retrieved from the RGAP database has 3, 758 base pairs (bp) structured into two UTRs, three exons and two introns (**Figure 1A**). The open reading frame (ORF) is 1, 431 bp, encoding a 476 amino acid polypeptide with an isoelectric point (pI) of 8.13 and a molecular weight of 50.1 kDa. To explore the evolutionary relationship between OsIDD9 and other IDD proteins, we constructed a phylogenetic tree using IDD family protein sequences of rice and *Arabidopsis* proteins (**Figure 1B**). The phylogenetic tree is divided into two distinct groups (Group I and Group II), the number of Group I members is more than Group II. OsIDD9 is in the Group I and closely related to AtIDD7, AtIDD11, OsIDD2, and OsIDD11, suggesting they have similar protein sequence.

### Production of *OsIDD9* CRISPR/Cas9 mutant and *OE* lines

**Figure 2:**
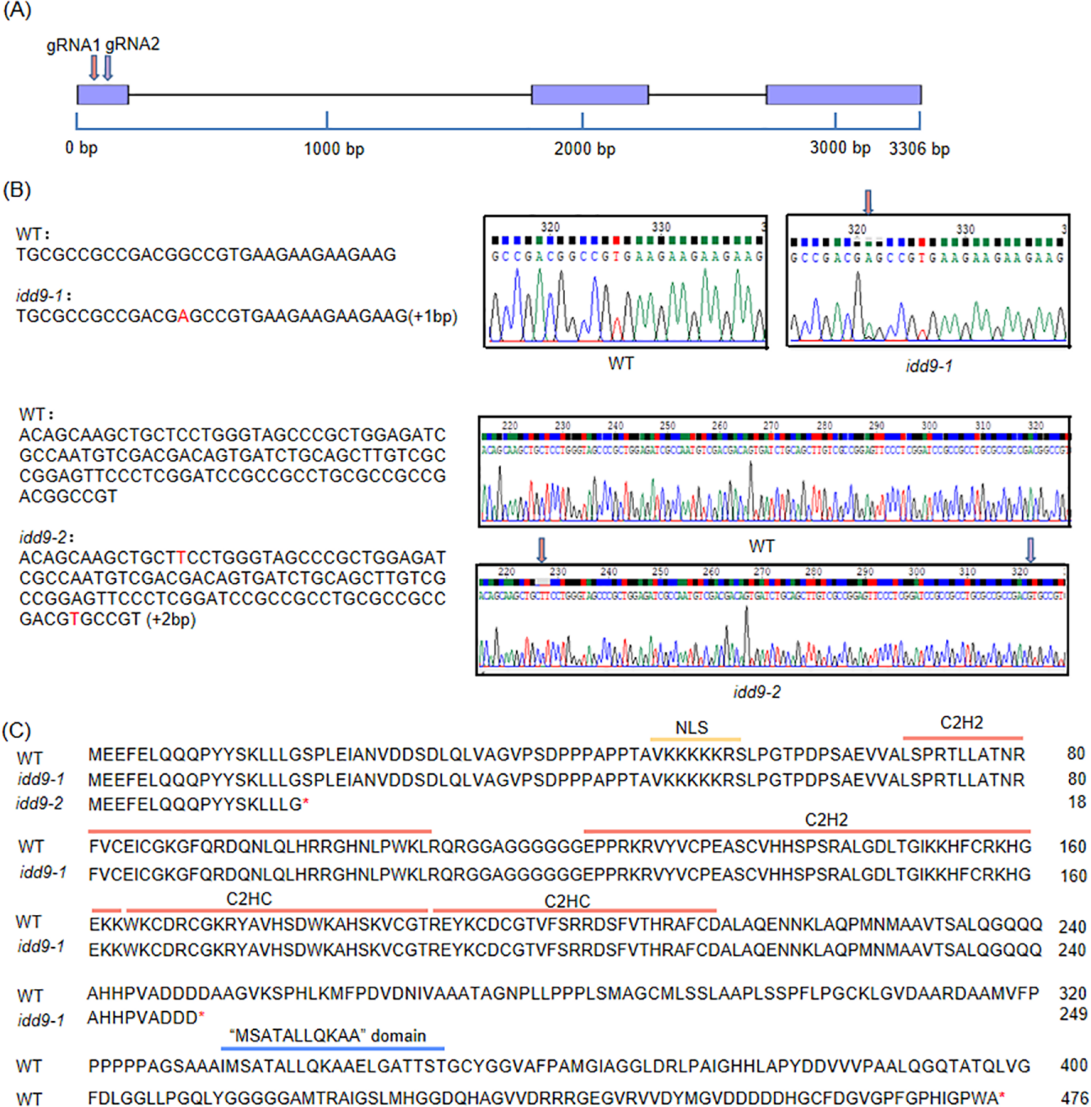
Generation of *OsIDD9* knockout mutants. **(A)** Diagram of *OsIDD9* gene structure. Yellow and green blocks represented the untranslated regions (UTRs) and exons, respectively. Black lines indicated introns. The arrows indicated CRISPR/Cas9 target sites, which were located inside the 1st exon. **(B)** Comparison of nucleotide sequences surrounding the gRNA sites of *OsIDD9* mutants and the wild-type (WT). Two independent alleles of *idd9* mutants (*idd9-1* and *idd9-2*) were generated using CRISPR/Cas9 gene-editing technology. The *idd9-1* and *idd9-2* had 1-bp and 2-bp insertion, respectively. The original positions of inserted bases were indicated by arrows in the corresponding chromatograms. **(C)** The protein sequence of OsIDD9 in the mutants compared with the WT sequence. The amino acids in the different domains were marked with a yellow, red, and blue line, respectively. Amino acids identical to the WT sequence were used in black. The termination of the translation was represented by the red “*”.

To study the function of *OsIDD9*, knockout mutants (*idd9*) and over-expression (*OE*) lines were produced. Clustered regularly interspaced short palindromic repeats (CRISPR)/CRISPR-associated protein 9 (Cas9) technology was used to create *idd9*. Two sites in the 1^st^ exon were selected for guide RNA (gRNA) design (**Figure 2A**) and constructed as described in the “Materials and methods” section. Among the mutant lines isolated, *idd9–1* has a single-nucleotide insertion (A insertion) at +142 bp, while the *idd9–2* mutant has two insertions, one T insertion at +48 bp and another T insertion at +142 bp (**Figure 2B**). Both mutations in *idd9–1* and *idd9–2* caused premature stop codons and thus truncated proteins. According to the previous report of *OsIDD* gene family, OsIDD9 protein has several obvious structural domains (Zhang et al., 2020) as shown in **Figure 2C**. However, each mutation causes a frameshift in the *OsIDD9* coding sequence that disrupts translation by generating a premature stop codon. For *idd9-1*, the truncated proteins have 249 amino acids and still remain NLS (nuclear localization signal), C2H2, and C2HC domains. The corresponding proteins in *idd9–2* lacked all domains and only retain about 4% of the length of the OsIDD9 protein in WT.

**Figure 3:**
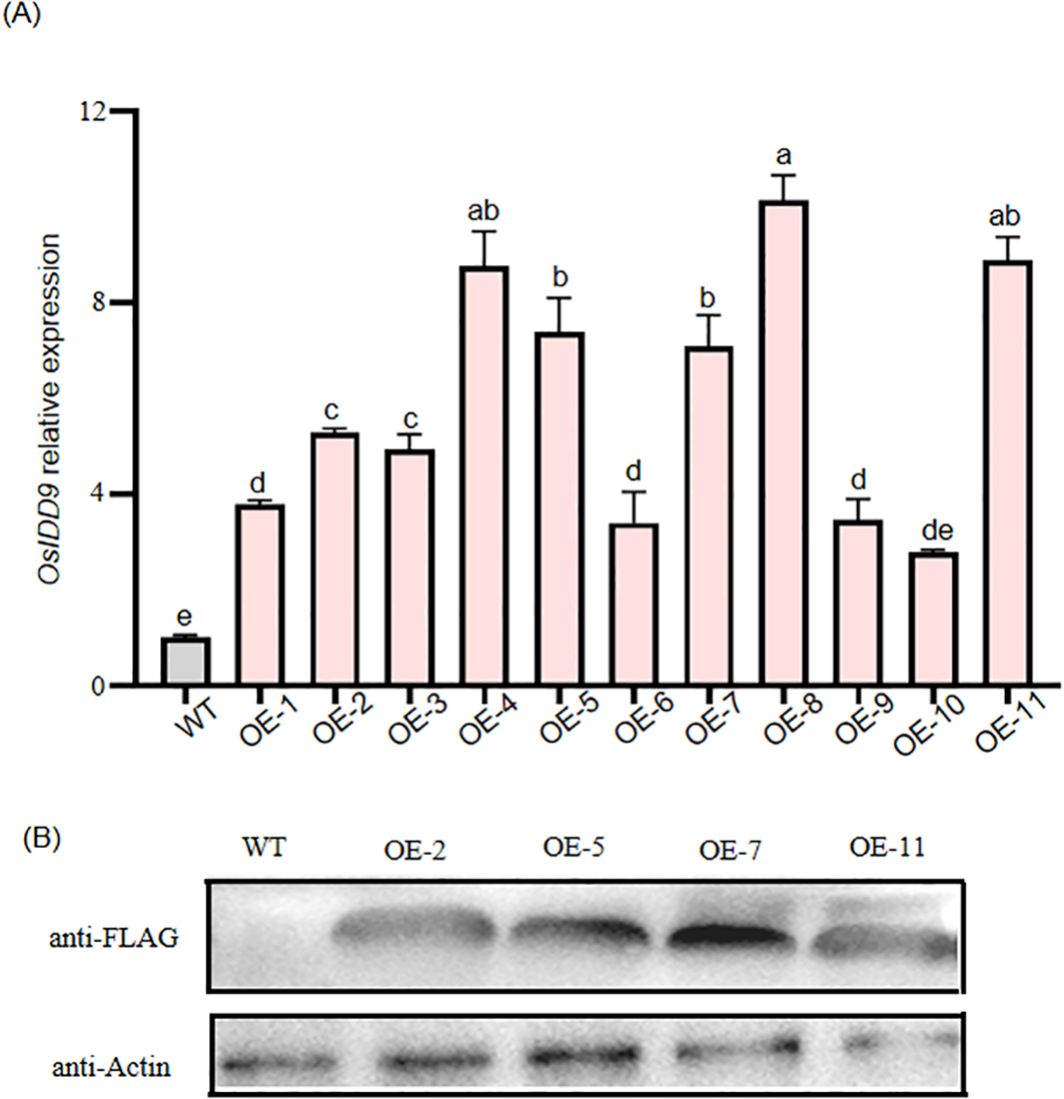
Generation of *OsIDD9* over-expression lines. **(A)** Detection of *OsIDD9* expression levels in WT and *OsIDD9* over-expression transgenic lines (*OE*) by reverse transcription quantitative polymerase chain reaction (RT-qPCR). *OsActin1* was used as the reference gene for normalization and the expression level of *OsIDD9* in WT was set to 1. Error bars represent ± SD of three biological replicates. One-way ANOVA was performed, followed by Tukey’s honestly significantly different (HSD) test. Different letters indicate statistically significant differences. **(B)** Immunoblot analysis using an anti-FLAG antibody confirmed expression of the OsIDD9-FLAG construct in the *OE* lines. Anti-actin (lower blot) was used as a loading control.

*OE* lines were produced by using a vector containing *OsIDD9* full length cDNA (complementary DNA) fused to the FLAG tag under the control of the ubiquitin promoter (*Ubi::OsIDD9-FLAG*) to transform ZH11 callus by Agrobacterium-mediated transformation. Eleven *OE* lines exhibited higher *OsIDD9* expression levels compared to WT (**Figure 3A**). Four lines (*OE-2*, *OE-5*, *OE-7*, and *OE-11*) were detected by Western Blot (**Figure 3B**) and three of them (*OE-2*, *OE-5*, and *OE-7*) were used for further study.

### *OsIDD9* is important for rice CR development

**Figure 4:**
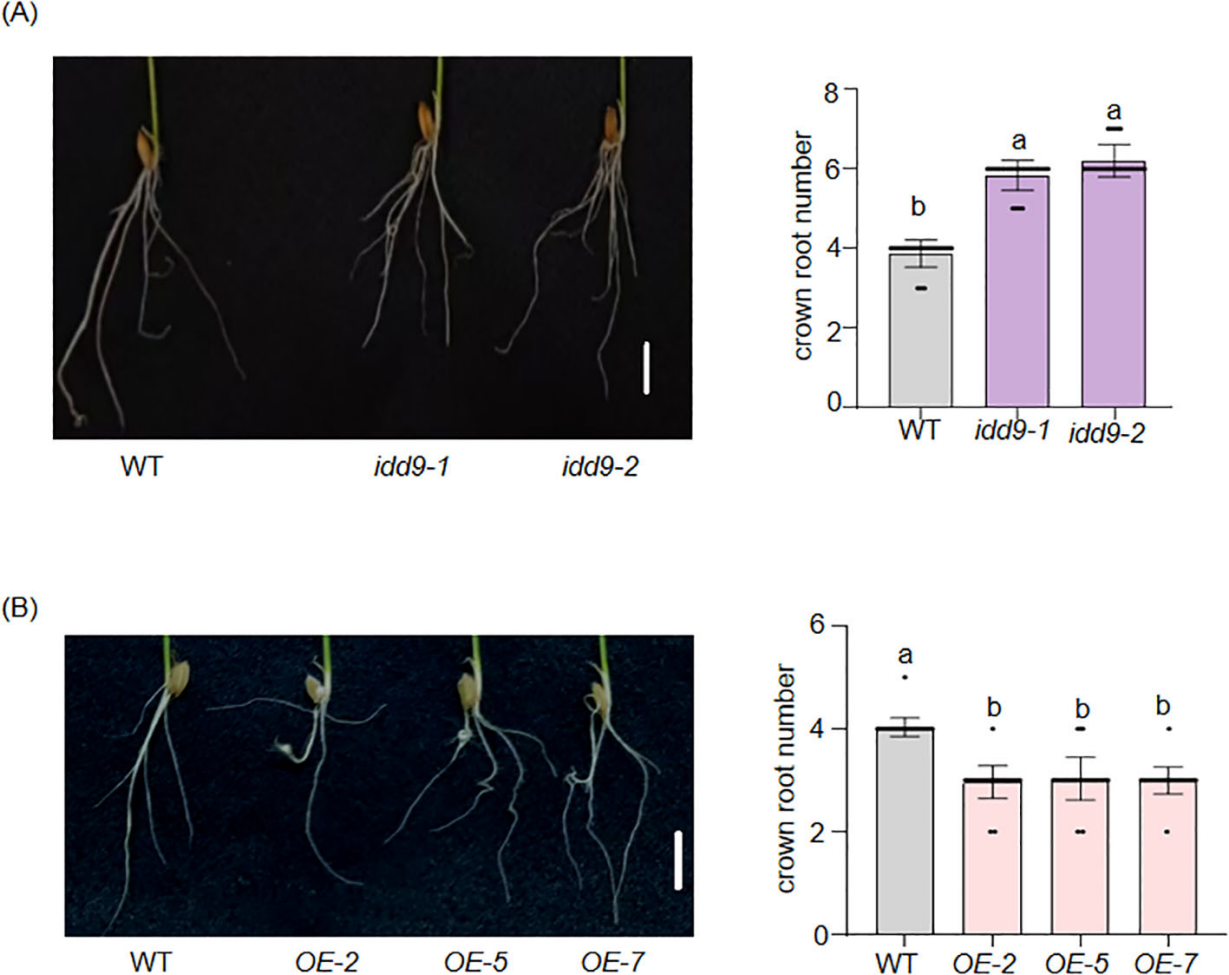
The expression of crown root development-related genes is regulated by OsIDD9. **(A)**Transcript levels of crown root development- related genes (*OsWOX11*, *OsCRL1*, *OsRR2*, *OsCKX4*, *OsCRL5*, and *OsLBD16*) in 7-day-old crown root of WT, *idd9*, and OE by RT-qPCR. *OsActin* was used as the reference gene for normalization and the gene transcript level in WT was set to 1.0. Data were shown as means ± SD from three biological replicates. One-way ANOVA was used to calculate P value. Different letters indicate statistically significant differences. **(B)** OsIDD9 inhibits the expression of *OsWOX11*. Schemes of the four constructs used in the co-transfection experiments of rice protoplasts. The promoter of *OsWOX11* was denoted by OsWOX11_P_ (left). OsIDD9 supressed the luciferase (LUC) expression under the control of OSWOX11_P_ in rice protoplasts (right). The diagrams of the constructs used in the co-transfection assays are presented on the left. Data were shown as means ± SD from three biological replicates. Student’s t-test was used to calculate P-value (**, P <0.01). **(C)** EMSA demonstrated OsIDD9 directly bound to *OsWOX11* *in vitro*. The His-tagged OsIDD9 C-terminal fusion protein (OsIDD9-His) was incubated with biotin-labeled DNA fragments, tested for competition by adding an excess of unlabeled probe (competitor). The probe sequences for EMSA are shown, with IDD binding motifs highlighted in red.

To determine whether *OsIDD9* can regulate rice root development, the expression levels of *OsIDD9* in different tissues were detected. The results were consistent with the previous report (Zhang et al., 2020), showing high expression in roots (**Supplementary Figure 1**). Subsequently, phenotypes of *idd9* and *OE* lines were examined. In the phenotypic analyses of WT and *idd9* (**Figure 4A**), WT has about four CRs (WT: 3.9 ± 0.34), the number of CRs in the *idd9* showed an increasing trend, with *idd9–1* having about six CRs (*idd9-1*: 5.8 ± 0.37) and *idd9–2* having about seven (*idd9-2*: 6.2 ± 0.40). The One-way ANOVA analysis shows that the *P* value between WT and *idd9–1* or *idd9–2* is less than 0.01, and the *P* between *idd9–1* and *idd9–2* is more than 0.05, suggesting that the differences in CR number of WT and *idd9* were significant. Meanwhile, The 1-week seedlings of *OE* had fewer CRs than WT (**Figure 4B**). When WT has around four CRs (WT: 4.0 ± 0.18), three *OE* lines all have about three CRs (*OE-2*: 2.96 ± 0.31; *OE-5*: 3.03 ± 0.41; *OE-7*: 3.00 ± 0.26), slightly lower than WT. These results demonstrate that *OsIDD9* plays a vital role in rice CR development.

### OsIDD9 can bind to the *OsWOX11* promoter and negatively regulates transcript level of CR development-related genes

**Figure 5:**
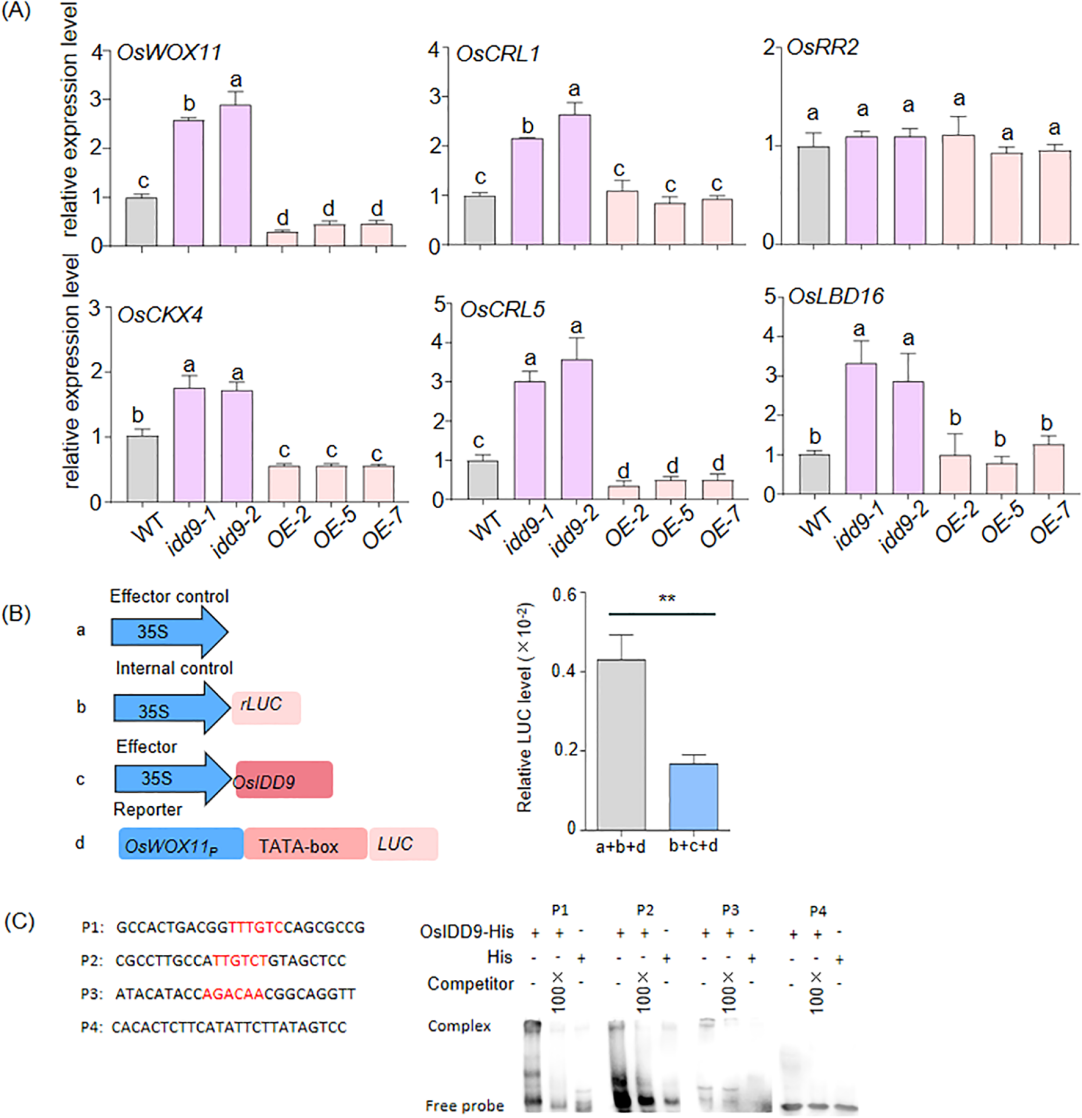
uORF_IDD9_-mediated reporter gene translation. **(A)** Sketch of *OsIDD9* structure. Yellow boxes represented UTRs and green box indicated CDS. The uORFs were located inside the 5’-UTR. **(B)** Schematic representation of 35S:uORF_IDD9_-LUC (uORF-LUC) and 35S:uorf_IDD9_-LUC (uorf-LUC) constructs to test whether the uORF_IDD9_ can repress mRNA translation in dual-luciferase system. Light and dark blue letters indicate the initiation and the stop codons of uORF1 and uORF2, respectively. Green letters represent the initiation codon of uORF3, which shares the stop codon with uORF1. Nucleotide mutations in the initiation and the stop codons of uORF_IDD9_ are indicated in red. **(C)** Effects of the uORF_IDD9_ and uorf_IDD9_ on LUC/REN activity and mRNA level in *Nicotiana benthamiana*. Data were shown as means ± SD from three biological replicates. Student’s t-test was used to calculate P-value (**P < 0.01). ns means no significant difference.

The vigorous crown root of *idd9* prompted us to examine the expression levels of CR development-related genes, such as *OsWOX11*, *OsCRL1*, *OsCRL5*, *OsRR2* (*A-type cytokinin response regulator 2*), *OsCKX4* (CYTOKININ OXIDASE/DEHYDROGENASE4), and *OsLBD16* (lateral organ boundaries domain16), via RT‐qPCR analysis using gene‐specific primer sets (**Supplementary Table 1**). *OsWOX11* and *OsCRL1* are the major factors affecting CR development, higher expression of *OsWOX11* will lead to more CRs, including the growth of ectopic roots, and if the *OsCRL1* is mutated, there will be no CR growth (Zhao et al., 2009; Kitomi et al., 2011; Dong et al., 2022). *OsWOX11* and *OsCRL1* act synergistically to regulate *OsCKX4* expression to maintain CR development (Geng et al., 2023). *OsLBD16* and *OsRR2* function as direct downstream factors of OsWOX11 in rice CR development (Zhao et al., 2015; Geng et al., 2024). As shown in **Figure 5A**, the expression levels of *OsCRL1*, *OsCKX4*, and *OsLBD6* were higher in *idd9* than in WT, however, the expression levels of these three genes in *OE* lines were comparable to those in WT. The expression level of *OsRR2* did not change significantly. The expression levels of *OsWOX11* and *OsCRL5* exhibited the same trend of variation in *idd9*, *OE* and WT. The expression levels of *OsWOX11* and *OsCRL5* in *idd9* were about three times higher than those in WT. While in *OE* lines, the expression levels were only about half of those in WT. These results suggest that OsIDD9 regulated CR development by repressing CR development-related gene expression.

To further explore the relationship between *OsWOX11* and OsIDD9, a dual-luciferase assay in rice protoplasts was executed and showed that OsIDD9 functions as a negative regulator of *OsWOX11* (**Figure 5B**). Previous studies reported the motifs that the IDD proteins bound in rice (RID1, 5’-TTTGTC-3’) (Wu et al., 2008) and in Arabidopsis (AtIDD3, 5’-AGACAA-3’) (Yoshida et al., 2014). The analysis of the 2.8-kb promoter sequence revealed that *OsWOX11* contains three putative IDD binding motifs. They were located 2716 to 2721 (P1), 1545 to 1550 (P2), and 1032 to 1037 (P3) bp upstream of the TSS (**Supplementary Figure 2**). Electrophoretic mobility shift assay (EMSA) showed that the OsIDD9-His fusion protein expressed in *E.coli* bound directly to P1, P2 and P3 fragments with IDD binding motifs, not to P4 (**Figure 5C**). In addition, unlabeled DNA fragment (competitor) inhibited binding (**Figure 5C**). These data suggested that OsIDD9 can bind to the *OsWOX11* promoter *in vitro*.

### *OsIDD9* has functional uORFs in its 5’UTR

**Figure 6:**
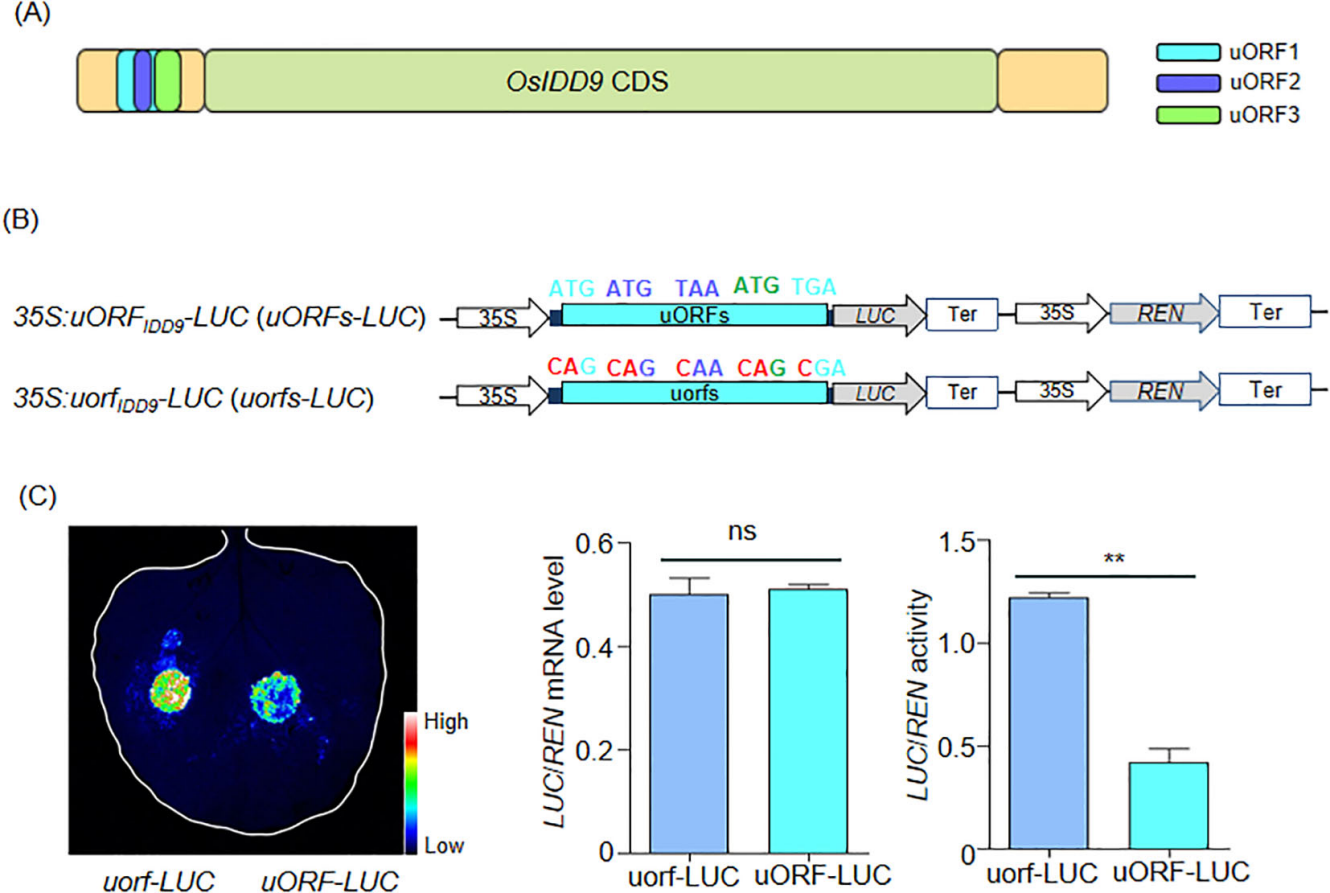
Function of *OsIDD9* in rice crown root development. **(A)** Crown root phenotypes of WT and *idd9* mutants at 7 days after germination. **(B)** Crown root phenotypes of WT and *OsIDD9* over-expression transgenic lines (*OE*) at 7 days after germination. Error bars were means ± SD (n = 30) from three biological replicates. Bars = 1 cm. One-way ANOVA was performed, followed by Tukey’s honestly significantly different (HSD) test. Different letters indicate statistically significant differences.

The expression of *OsIDD9* has been reported to be regulated by hormones and abiotic stresses (Zhang et al., 2020), but whether it is controlled in other ways is elusive. uORF is an important regulatory element, which can decrease the translation level of mORF (major open reading frames) located downstream of it (Um et al., 2021; Wang et al., 2024). In order to find out whether *OsIDD9* has uORFs, the 5’UTR sequence of *OsIDD9* was screened by ORF finder in NCBI. Three uORFs were found in the 5’UTR of *OsIDD9* and located +72 to +185, +101 to +133, and +138 to +185, respectively (**Figure 6A**). To investigate whether these uORFs play an important role in translation regulation, the construct of these uORFs fused firefly luciferase (LUC) driven by a CaMV 35S promoter (*uORFs-LUC*) was generated. As a control, 35S:uorfs-LUC construct (*uorfs-LUC*) was also generated in which that the initiation and the stop codons of uORFs were mutated (ATG→CAG, TGA→CGA, and TAA→CAA) (**Figure 6B**). Both constructs were then transiently transformed into tobacco leaves to quantify the expression of *LUC*. LUC activity was apparently repressed in the *uORFs-LUC* line compared to that in *uorfs-LUC* (**Figure 6C**). However, qRT-PCR analysis revealed no significant differences in the levels of *LUC*/*REN* mRNA transcribed from the *uORFs-LUC* and *uorfs-LUC* lines (**Figure 6C**). These data indicate that uORFs of *OsIDD9* regulates the translation of downstream proteins without changing their mRNA levels, and they may be functional regulatory elements.

## Discussion

IDD proteins are a class of plant-specific TF family, which have a broad range of activities in the formation of various plant tissues and organs, as well as throughout plant development. Bioinformatics analysis has indicated that IDD proteins in all land plants have been divided primarily into two groups: Group I and Group II (**Figure 1B**). Group II, represented by AtIDD14, AtIDD15, and AtIDD16, mainly controls in the morphogenesis of aerial organs and gravitropic responses (Cui et al., 2013). The functions of the *IDD* genes in Group I have diversified during the land plant evolution (Coelho et al., 2018; Kozaki, 2024). To date, most of the *IDD* genes in Group I that have been mainly characterized are from *Arabidopsis*. However, a few have been functionally reported in rice. They are involved in flowering time, cold stress, nitrogen absorption and may have a direct or indirect impact on yield (Wu et al., 2008; Xuan et al., 2013; Dou et al., 2016; Deng et al., 2017). Based on these, more information about the functions of OsIDDs will provide more valuable insights into the signaling network in rice development and metabolic regulation. Compared to other members in Group I, OsIDD9 lacks a domain (“TRDFLG” domain) (Zhang et al., 2020), which was verified to be related with protein-protein interaction (Colasanti et al., 2006). This suggests that OsIDD9 may have functional divergence from other members. Here, phenotypic studies showed that *osidd9* knockout mutants generated by CRISPR-cas9 exhibited more and shorter crown roots than WT in 1-week-old seedlings (**Figure 4A**). By contrast, *OE* lines displayed a defective CR phenotype. The number of CRs was correspondingly decreased in three *OE* lines than WT (**Figure 4B**). In addition, the expression levels of some known genes affecting CR development showed significant differences between the knockout mutants and *OE* lines (**Figure 5A**). Together, these results demonstrated that OsIDD9 is involved in rice CR development by regulating other CR development-related gene expression.

In the regulatory network of rice CR development, quite a few TFs affecting CR development have been identified. Because the development of rice CR is a coordinated process of multi-genes, it is important to clarify the regulatory relationship between these factors. As a key regulatory factor, OsWOX11 plays a central role in rice CR development (Zhao et al., 2009). On one hand, OsWOX11 can interact with OsCRL1, which is considered as a pioneer factor for CR development, to promote *OsCKX4* expression (Geng et al., 2023). OsWOX11-ERF3 interaction suppresses *OsRR2* expression (Zhao et al., 2015). OsWOX11 can also recruit some epigenetic modifiers such as GCN5 and JMJ706 to active the expression of downstream genes (Zhou et al., 2017). On the other hand, OsWOX11 integrates the auxin, cytokinin and ROS signaling pathways during different stages of CR development (Jiang et al., 2017). Overall, the regulatory network of CR development centered on OsWOX11 is complicated. In comparison, the upstream factors of *OsWOX11* in this regulatory network have been rarely reported. Only OsEIL1 (ETHYLENE INSENSITIVE 3-LIKE 1) or OsHsfA1a (Heat shock factor A1) have been reported as its upstream activators (Li et al., 2024; Zhang et al., 2025a). In this study, the increased expression of *OsWOX11* was observed in *osidd9* mutants, and the declined expression in *OE* lines (**Figure 5A**). Moreover, OsIDD9 can directly binds to the *OsWOX11* promoter and regulate its expression (**Figures 5B, C**). These indicated that OsIDD9 was likely the direct negative factor of *OsWOX11*. Apart from *OsRR2*, the expression levels of other *OsWOX11*-related developmental factors in the *osidd9* mutants also showed variations, suggesting that OsIDD9 might regulate rice CR development through the verified *OsWOX11* regulatory pathway.

As an important translation regulatory element, uORF has been reported to be involved in plant nutrient uptake, stress response and metabolic regulation (Tanaka et al., 2016; Xu et al., 2017; Reis et al., 2020; Yang et al., 2020; Nguyen et al., 2023; Tian et al., 2024; Sang et al., 2025; Zhang et al., 2025b).There are also a few uORFs that have been shown to be related to plant growth and development (Liu et al., 2021; Yang et al., 2024; Wang et al., 2025; Zhang et al., 2025a). For example, the absence of uORF_OsHd2_ can cause to delay rice heading date (Liu et al., 2021), uORF_OsDEP1_ and uORF_OsGIF1_ are related to rice yield, and uORF_OsHsfA1a_ control crown root architecture (Zhang et al., 2025a). Similarly, three uORFs were also found in the 5′UTR of *OsIDD9*. Luciferase-based reporter assays revealed that uORF_IDD9_ remarkably repressed the expression of the reporter gene (**Figure 6**). It is worth further exploring whether the uORF_IDD9_ can regulate the development of CRs. Collectively, our data enriched the function of IDD members and identified OsIDD9 as a new upstream factor of OsWOX11-dependent CR development. Future analysis aimed at dissecting the translation regulation of OsIDD9 may provide new insights.

## Data Availability

The original contributions presented in the study are included in the article/**Supplementary Material**. Further inquiries can be directed to the corresponding author.

## Supplementary material

Supplementary Figure 1 Expression analysis of OsIDD9 in various tissues detected by RT-qPCR. OsACTIN was used as the internal standard. For statistical analysis, one-way ANOVA was performed followed by Tukey’s honestly significantly different (HSD) test. Different letters indicated statistically significant differences.

Supplementary Figure 2 Sketch of OsWOX11 structure. UTRs and exons were represented by yellow and green boxes, respectively. The promoter of OsWOX11 was shown by the black line. Three putative IDD binding motifs (P1-P3) in the OsWOX11 promoter were indicated on each by pink vertical lines.
