# Supplementary material for: INDETERMINATE DOMAIN 9 negatively regulates rice crown root development
Source: Front Plant Sci. 2026 Apr 13;17:1820594. doi: 10.3389/fpls.2026.1820594 (PMC13111443; doi:10.3389/fpls.2026.1820594)
Supplement: Supplementary Figure 1 — Expression analysis of OsIDD9 in various tissues detected by RT-qPCR. OsACTIN was used as the internal standard. For statistical analysis, one-way ANOVA was performed followed by Tukey’s honestly significantly different (HSD) test. Different letters indicated statistically significant differences. [file Presentation1.pptx]

## Slide 1
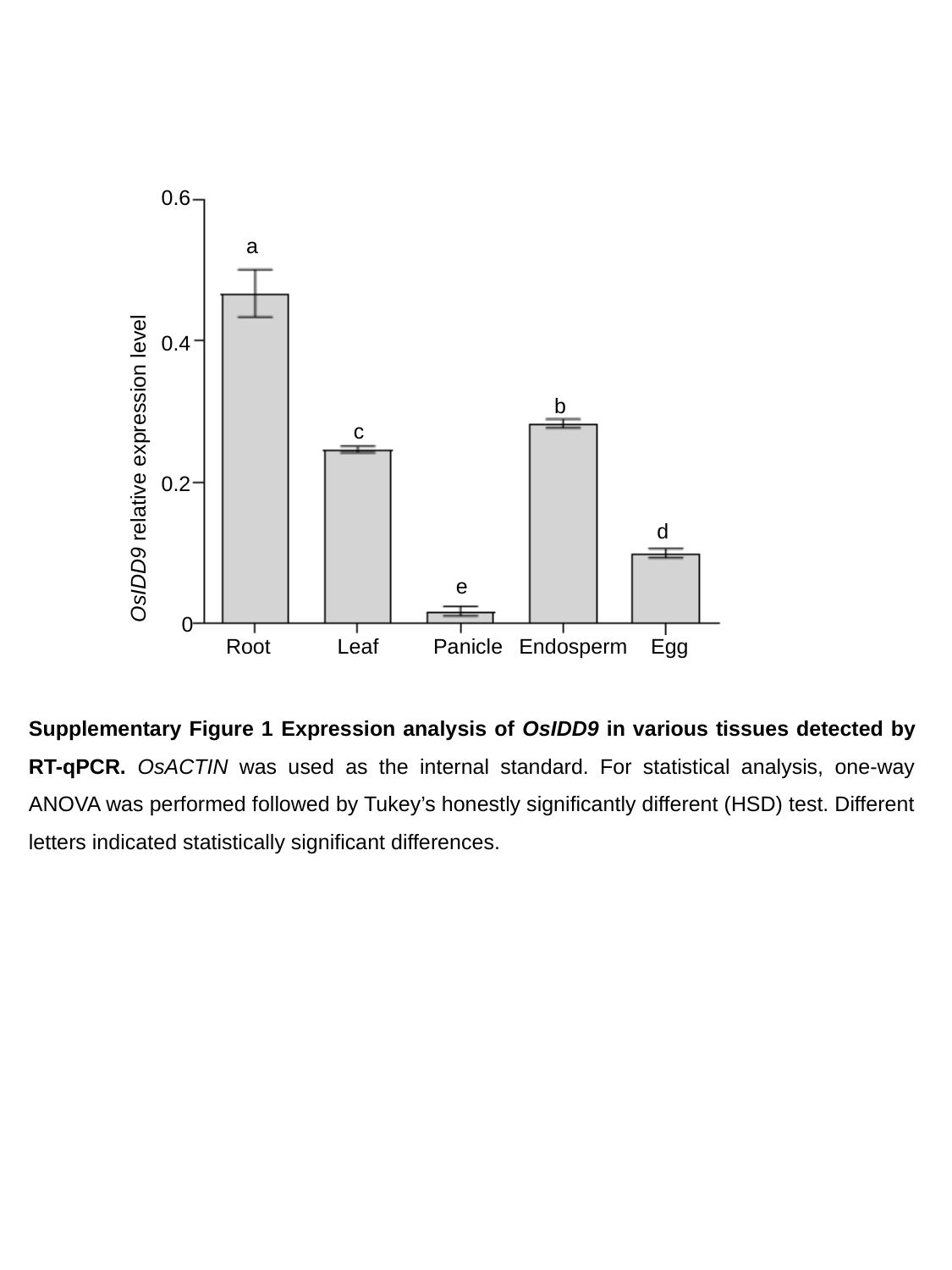

0.6
a
0.4
b
OsIDD9 relative expression level
c
0.2
d
e
0
Root
Leaf
Panicle
Endosperm
Egg
Supplementary Figure 1 Expression analysis of OsIDD9 in various tissues detected by RT-qPCR. OsACTIN was used as the internal standard. For statistical analysis, one-way ANOVA was performed followed by Tukey’s honestly significantly different (HSD) test. Different letters indicated statistically significant differences.

## Slide 2
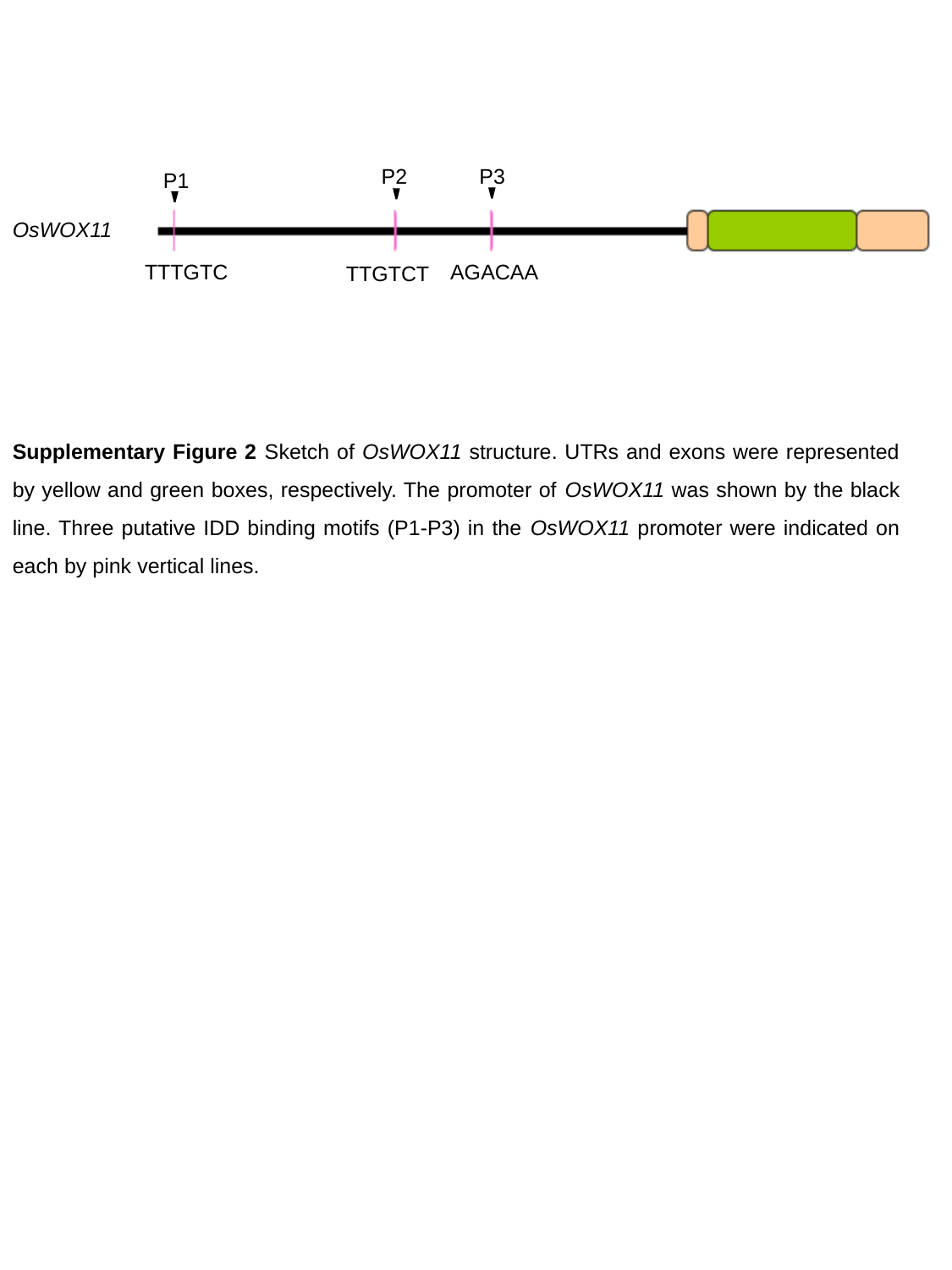

P2
P3
P1
OsWOX11
TTTGTC
AGACAA
TTGTCT
Supplementary Figure 2 Sketch of OsWOX11 structure. UTRs and exons were represented by yellow and green boxes, respectively. The promoter of OsWOX11 was shown by the black line. Three putative IDD binding motifs (P1-P3) in the OsWOX11 promoter were indicated on each by pink vertical lines.
